# The magnitude of the sound-induced flash illusion does not increase monotonically as a function of visual stimulus eccentricity

**DOI:** 10.3758/s13414-022-02493-4

**Published:** 2022-05-13

**Authors:** Niall Gavin, Rebecca J. Hirst, David P. McGovern

**Affiliations:** 1 School of Psychology, Dublin City University, Glasnevin Campus, Dublin 9, Ireland; 2 School of Psychology and Institute of Neuroscience, Trinity College Dublin, Dublin 2, Ireland

**Keywords:** Sound-induced flash illusion, Multisensory integration, Audiovisual integration, Temporal binding window, Eccentricity

## Abstract

The sound-induced flash illusion (SIFI) occurs when a rapidly presented visual stimulus is accompanied by two auditory stimuli, creating the illusory percept of two visual stimuli. While much research has focused on how the temporal proximity of the audiovisual stimuli impacts susceptibility to the illusion, comparatively less research has focused on the impact of spatial manipulations. Here, we aimed to assess whether manipulating the eccentricity of visual flash stimuli altered the properties of the temporal binding window associated with the SIFI. Twenty participants were required to report whether they perceived one or two flashes that were concurrently presented with one or two beeps. Visual stimuli were presented at one of four different retinal eccentricities (2.5, 5, 7.5, or 10 degrees below fixation) and audiovisual stimuli were separated by one of eight stimulus-onset asynchronies. In keeping with previous findings, increasing stimulus-onset asynchrony between the auditory and visual stimuli led to a marked decrease in susceptibility to the illusion allowing us to estimate the width and amplitude of the temporal binding window. However, varying the eccentricity of the visual stimulus had no effect on either the width or the peak amplitude of the temporal binding window, with a similar pattern of results observed for both the “fission” and “fusion” variants of the illusion. Thus, spatial manipulations of the audiovisual stimuli used to elicit the SIFI appear to have a weaker effect on the integration of sensory signals than temporal manipulations, a finding which has implications for neuroanatomical models of multisensory integration.

**Supplementary Information:**

The online version contains supplementary material available at 10.3758/s13414-022-02493-4.

To promote effective interaction with the environment, our brains are required to combine information arriving from multiple sensory modalities. When this sensory information is aligned in space and time, multisensory integration is of benefit to the observer leading to more precise sensory estimates and helping to create a coherent percept of the environment (Ernst & Banks, 2002; Ernst & Bulthoff, 2004). However, in cases where there are spatial or temporal discrepancies between the unimodal estimates, multisensory illusions can occur, with some classic examples including the ventriloquist effect, in which the perceived location of an auditory stimulus is biased towards the location of the visual stimulus (Alais & Burr, 2004; Howard & Templeton, 1966; McGovern et al., 2016), and the McGurk effect, where a conflict in auditory and visual speech components can result in the perception of an entirely different sound (Hirst et al., 2018; McGurk & MacDonald, 1976; Munhall et al., 1996).

While many of the classic multisensory illusions involve visual perception altering the perception of other sensory modalities, the sound-induced flash illusion (SIFI; Shams et al., 2000, 2002)—in which the presence of multiple auditory stimuli causes a single visual flash to appear as multiple flashes—provides a striking example of auditory information biasing visual perception. The discovery of the SIFI heralded a paradigm shift in the field of multisensory perception in that it provided evidence that, rather than vision simply dominating the other senses, the brain combines information between the senses in a near-optimal manner, whereby each unimodal estimate is weighted according to its reliability in a given context (e.g., Alais & Burr, 2004; Ernst & Banks, 2002; Shams et al., 2005). Since its discovery more than 20 years ago, the SIFI has been employed as a useful means to assess the nature of multisensory perception in humans and has helped to reveal, amongst other things, the influences of perceptual experience, ageing and a variety of clinical conditions on multisensory integration (see Hirst et al., 2020; Keil, 2020, for recent reviews).

One particularly useful application of the SIFI is as a tool for measuring the “temporal binding window” of multisensory integration, which describes the brain’s tolerance to time differences between unimodal sensory estimates. According to this view, sensory signals that are in close temporal proximity are combined or integrated into a single multisensory percept, while signals that are separated by longer durations remain segregated. To provide a measure of the temporal binding window using the SIFI, previous research has manipulated the stimulus onset asynchronies (SOAs) of the audiovisual stimuli with the results showing that the susceptibility in experiencing the SIFI reduces as the temporal proximity of the audiovisual stimuli is decreased (e.g., McGovern et al., 2014; Setti et al., 2011; Shams et al., 2000). A similar effect is also observed for the less-studied fusion variant of the SIFI where two flashes are perceived as being one flash when presented with a single beep (e.g., McGovern et al., 2014). Thus, the SIFI provides a useful means for measuring the temporal binding window that accords well with other measurement methods (see Stevenson et al., 2012).

While manipulating the temporal proximity of audiovisual stimuli has a systematic effect on SIFI susceptibility, the impact of spatial manipulations of the stimuli are less clear. For instance, two studies found no impact of spatial disparity between auditory and visual stimuli on susceptibility to either the fission or fusion form of the SIFI (DeLoss & Andersen, 2015; Innes-Brown & Crewther, 2009). On the other hand, three studies provided evidence for greater fission susceptibility when the visual stimulus occurred in the periphery of the visual field at 5 degrees (Tremblay et al., 2007), 8 degrees (Shams et al., 2001) and 10 degrees (Chen et al., 2017) eccentricity when compared to foveal presentation. Meanwhile, there have been mixed findings on differences in fusion SIFI susceptibility depending on the visual stimulus location, with one study finding no differences in susceptibility between presenting the visual stimulus centrally compared to peripherally at 5 degrees (Tremblay et al., 2007), while another found susceptibility to be greater when the visual stimulus was presented centrally compared to peripherally at 10 degrees (Chen et al., 2017).

While these studies provide useful insights into the impact that spatial configurations of audiovisual stimuli have on susceptibility to the SIFI, a number of important questions remain. For instance, a consistent finding in the existing literature is that the fission illusion is stronger in the peripheral visual field relative to the fovea (Chen et al., 2017; Shams et al., 2001; Tremblay et al., 2007), which may be explained by the larger number of neuroanatomical connections between auditory and visual cortices in the periphery (see Falchier et al., 2002; Rockland & Ojima, 2003). However, if enhanced connectivity between auditory and visual cortices can explain the differences in the strength of the SIFI in peripheral and central fields, one might also expect susceptibility to the fission illusion to increase systematically with increasing visual stimulus eccentricity given that Falchier et al. (2002) demonstrated that the number of projections from auditory cortex to area 17 increased monotonically as function of eccentricity, although this has yet to be tested. Furthermore, given that most of the studies that have examined the effect of eccentricity on the SIFI have only used one SOA between the auditory and visual stimulus, the impact of visual stimulus eccentricity on the shape of the temporal binding window is unclear. There are at least three different ways in which manipulating the position of the visual stimulus could affect the temporal binding window; it could lead to a narrowing of the binding window, a reduction in its peak amplitude or a combination of both (Fig. 1). Based on the existing literature, it might be expected that the amplitude of the temporal binding window associated with the fission SIFI would increase with greater eccentricity (Chen et al., 2017; Shams et al., 2001; Tremblay et al., 2007; however, the impact of eccentricity on the width of the temporal binding window is less clear.

**Fig. 1:**
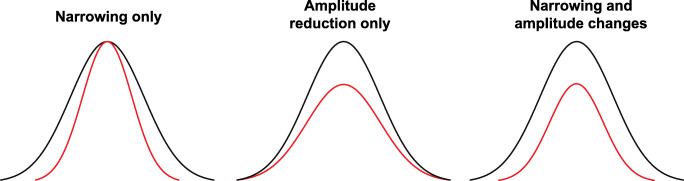
Schematic illustrating potential ways that the shape of the temporal binding window can be altered via narrowing alone, a reduction in the peak amplitude alone, or both together

The current study aimed to address these gaps in the literature by systematically manipulating the retinal eccentricity of the visual stimulus (2.5, 5, 7.5 and 10 degrees), as well as the SOAs of the audiovisual stimuli (eight values ranging −400 to 400 ms). Thus, the study set out to assess not just whether stimulus eccentricity affects overall susceptibility to the SIFI, but also whether it has an impact on how participants combine auditory and visual information in different temporal contexts. Based on the previous literature we hypothesized that participants would exhibit a graded increase in susceptibility to the fission SIFI as the visual stimulus was presented further into the periphery, and that this would be reflected as an increase in the peak amplitude of the temporal binding window. Given the mixed findings regarding the effect of eccentricity on the fusion SIFI, it was less clear what to expect for this variant of the illusion, but we included it in our analysis to expand our understanding of possible parallels and differences between fission and fusion illusions.

## Methodology

### Participants

Twenty participants (nine female, age range: 18–40 years, mean age = 24.9 years) volunteered to take part in the study. An *a priori* power analysis was not conducted, but a sample size of twenty was judged to be reasonable given existing literature showing a robust effect of SOA on SIFI susceptibility and given the large number of trials completed by each participant (trials per participant = 1,920). Furthermore, frequentist statistics were paralleled with Bayesian analyses to ensure that null effects provided genuine support for the null and did not reflect underpowered statistical tests. Criteria for inclusion were normal or corrected-to-normal vision and hearing; no previous history of flash sensitivity, epilepsy or migraines. Due to travel restrictions imposed by the COVID-19 pandemic, participants were recruited from the first-author’s neighbourhood (N.G.) and completed the experiment in a dedicated testing room in the author’s house. Participants did not receive a gratuity for their participation. All recruitment and experimental procedures were approved by the School of Psychology Research Ethics Committee, Dublin City University.

### Apparatus and stimuli

Auditory and visual stimuli were presented on a MacBook Pro laptop and programmed in PsychoPy (Version 2020.1.0; Peirce, 2007, 2009). The visual stimulus took the form of a briefly presented (17 ms) circular disk stimulus (i.e., a flash) with a diameter of 1 degree. The auditory stimulus was a briefly presented (17 ms) “beep” sound with a frequency of 3.5 kHz presented via Sennheiser HD 215 headphones at a sound pressure level of 70 dB. Participants viewed the visual stimuli with their head supported by a chin rest positioned 57 cm away from an LCD screen (refresh rate: 60 Hz, spatial resolution: 2,560 × 1,600 pixels), while fixating on a fixation cross 2 degrees from the top of the LCD screen. Depending on the trial, the visual stimulus could be presented 2.5, 5, 7.5, or 10 degrees below the fixation cross.

### Procedure

Participants were presented with brief visual flashes alongside auditory beeps and were required to indicate whether they saw one or two flashes by pressing a button on a keyboard (the ‘1’ key for one flash and the ‘2’ key for two flashes). Participants were instructed to ignore any beeps they heard while performing the task. To familiarize participants with the task and to ensure that they were satisfactorily maintaining fixation on the fixation cross, participants were given five practice trials before beginning the main experiment.

**Fig. 2:**
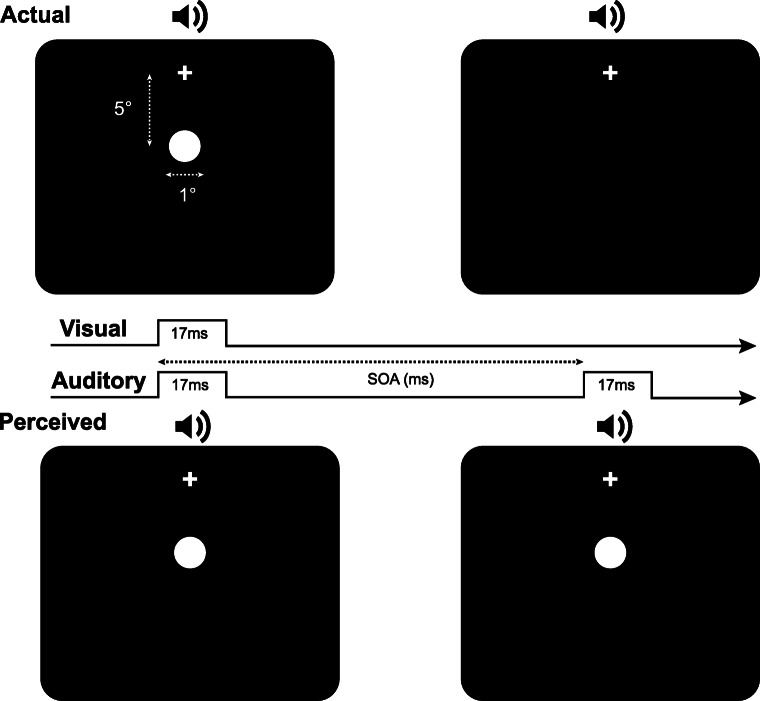
A schematic example of a 1F2B trial, with a positive SOA (i.e., visual lead), in which one visual flash is presented with two auditory beeps (upper part of figure) that gives rise to the perception of two flashes (lower part of the figure)

On each trial participants were presented with one or two visual flashes accompanied by one, two or no auditory beeps. Thus, for each different eccentricity condition there were six different “types” of trial, representing all possible combinations of flashes and beeps (1F2B, 2F1B, 2F2B, 1F1B, 2F0B, 1F0B). For convenience, these trial types are represented by an abbreviation that relates to their veridical percept. For example, trials described as 1F2B refer to trials where one flash was accompanied by two beeps, while 2F0B indicates trials containing two flashes and no beeps. On trials involving more than one flash or one beep, the auditory and visual stimuli were separated by different SOAs (differences in the presentation time of the first and second sets of stimuli; see Fig. 2), which could take one of eight values (−400, −200, −100, −50, 50, 100, 200, 400 ms). On trials designed to induce the SIFI (i.e., 1F2B trials), positive (negative) values indicated conditions where the second beep was presented after (before) a simultaneous flash-beep pair. To avoid the creation of unintentional response biases, there was an equal number of trials for each of the different trial types, such that trials were added for conditions where SOA was not manipulated due to there being only one stimulus presentation (i.e., 1F0B, 1F1B). Thus, altogether the stimulus conditions consisted of four visual stimulus eccentricities, six trial types and eight stimulus onset asynchronies combining to form 192 different conditions. Participants completed at least 10 trials per condition leading to a total of 1,920 trials per participant (total trial count across participants = 38,400) with all trials randomly interleaved for each participant (i.e., the eccentricity of the visual stimulus varied from trial to trial). At regular intervals over the course of the experiment (every 192 trials), participants were prompted to take a self-timed break to avoid fatigue. In total, the experiment lasted approximately 75–80 minutes. Throughout the experiment, the experimenter observed the participant from a distance to ensure that they maintained their gaze on the fixation cross.

### Data analysis

Statistical analyses consisted of separate two-way repeated-measures ANOVAs to analyze the effects of stimulus eccentricity and stimulus onset asynchrony on the proportion of illusory responses as well as the interaction between these variables for 1F2B and 2F1B trials. To establish the strength of evidence behind the presence or absence of any observed effects, the data were also analyzed with Bayesian methods through JASP (JASP Team, 2020). A Bayes factor analysis overcomes some of the issues associated with null hypothesis testing by quantifying the relative likelihood of the data under the null and alternative hypothesis (Dienes, 2014). All Bayesian analyses of variance used the default settings in JASP (r scale fixed effects = 0.5; r scale random effects = 1; r scale covariates = 0.354). We interpret the resulting Bayes factors in line with recommendations by Dienes (2014), whereby a BF_10_ of 3 or over indicated moderate-strong support for the alternative hypothesis, while a BF_10_ lower than 0.3 provided moderate-strong support for the null hypothesis. Values that fell within the range of 1–3 provided weak or inconclusive support for the alternative hypothesis, while values between 0.3 and 1 provided weak support for the null hypothesis (Dienes, 2014).

To assess the impact of visual stimulus eccentricity on the width and peak amplitude of the temporal binding window, a Gaussian curve was fitted to both the group-averaged data and to the data of individual participants where the standard deviation and amplitude of the Gaussian were left as free parameters. To ensure that the parameter values derived from the Gaussian fits to the individual data provided meaningful estimates of the temporal binding window, curve fits that produced an *R*^2^ of less than 60% were excluded from this analysis. This led to the removal of five participants from the analysis of the fission data and three participants from the fusion analysis. The data from these participants were included in the fits to the group-averaged data, as well as the repeated-measures ANOVAs described in the preceding paragraph.

### Data availability

The datasets analysed during the current study are available at https://osf.io/mnc9g/.

## Results

### Fission SIFI

**Fig. 3:**
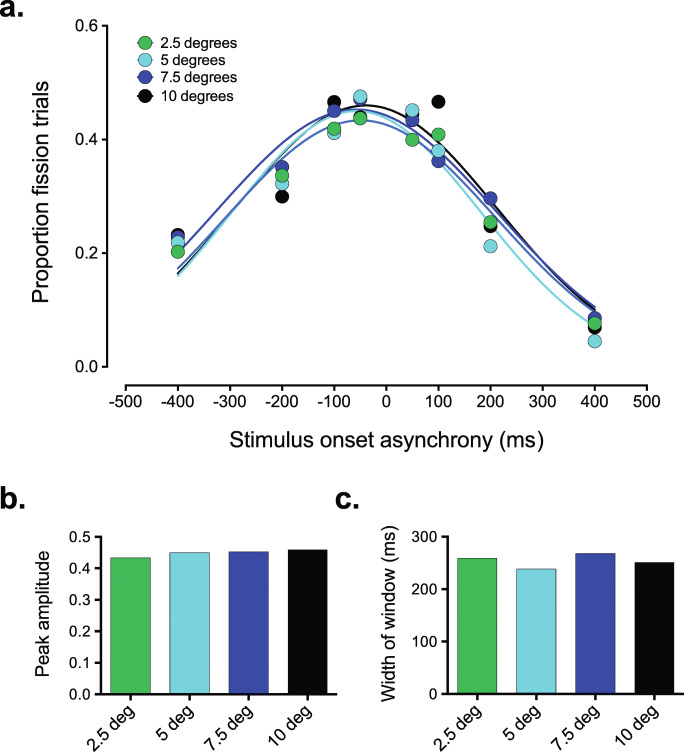
Visual stimulus eccentricity has little effect on the shape of the temporal binding window associated with the fission SIFI. **a** While increasing the temporal difference between the audiovisual stimuli led to a systematic decrease in the magnitude of the SIFI, the effect of visual stimulus eccentricity was much smaller. **b** Fitting the group-averaged data with a Gaussian curve revealed a subtle increase in the peak amplitude of the temporal binding window (2.5 deg.: 0.433, 5 deg.: 0.45, 7.5 deg.: 0.453, 10 deg.: 0.46). **c** No systematic effect of visual stimulus eccentricity on the width of the temporal binding window was observed (2.5 deg.: 259 ms, 5deg.: 238 ms, 7.5 deg,: 268 ms, 10 deg.: 251 ms)

To assess the impact of visual stimulus eccentricity on the temporal binding window associated with the fission SIFI, a two-way repeated-measures ANOVA was conducted with visual stimulus eccentricity and stimulus onset asynchrony as factors. As expected, and in line with previous studies (e.g., McGovern et al., 2014; Setti et al., 2011; Shams et al., 2000), there was a significant effect of SOA on fission susceptibility, *F*(7, 133) = 23.46, *p* < .001, partial η^2^ = 0.55, BF_10_ = 2.322e +61, such that participants were less likely to experience the illusion with larger SOAs. However, there was no significant effect of eccentricity on susceptibility to the fission illusion, *F*(3, 57) = 0.9, *p* = .45, partial η^2^ *=* 0.045, BF_10_ = 0.01 (see Fig. 3a). The interaction between SOA and stimulus eccentricity was also non-significant, *F*(21, 399) = 1.057, *p* = .393, partial η^2^ *=* 0.053, BF_10_ = 0.001, indicating that the influence of changes in SOA on fission susceptibility did not depend on changes in eccentricity, nor vice versa.

To more accurately quantify the impact of visual stimulus eccentricity on the temporal binding window, a Gaussian curve was fitted to the group-averaged data for each eccentricity condition, with the amplitude and standard deviation of the Gaussian function fit taken as estimates of the peak amplitude and width of the temporal binding window, respectively. The parameter estimates for the curve fits to the group-averaged data are summarized in Fig. 3 and suggest that there may be a small but systematic increase in the peak amplitude of the temporal binding window with increasing eccentricity of the visual stimulus (Fig. 3b), although no systematic changes were observed the width of the temporal binding window (Fig. 3c). To assess whether the change in the amplitude of the temporal binding window as a function of visual stimulus eccentricity was statistically significant, we also fitted the Gaussian function to the individual fission data to estimate the peak amplitude and standard deviation for each participant (see Data Analysis for further details on this procedure). A one-way repeated-measures ANOVA conducted on the individual measures of the peak amplitude showed that the eccentricity of the visual stimulus had no impact on susceptibility to the fission illusion, *F*(3, 42) = 0.112, *p* = .953, partial η^2^ *=* 0.008, BF_10_ = 0.1. There was also no significant effect of eccentricity on the width of the temporal binding window, *F*(3, 42) = 2.11, *p* = .114, partial η^2^ *=* 0.13, although this only met the criterion for weak evidence in support of the null (BF_10_ = 0.73).

### Fusion SIFI

**Fig. 4:**
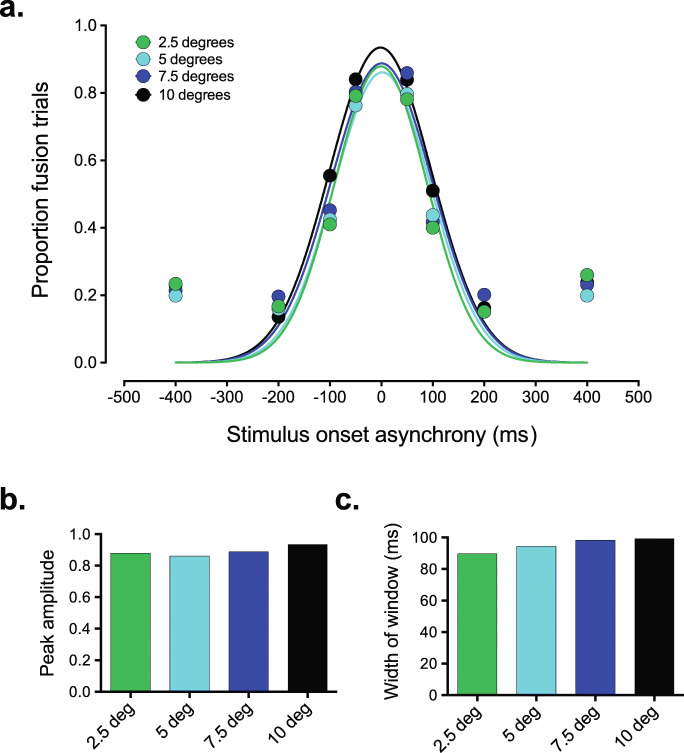
The shape of the temporal binding window as a function of visual stimulus eccentricity associated with the fusion SIFI. **a** While increasing the temporal difference between the audiovisual stimuli led to a systematic decrease in the magnitude of the fusion SIFI, the effect of visual stimulus eccentricity was much smaller. **b** Fitting the group-averaged data with a Gaussian curve revealed a subtle increase in the peak amplitude of the temporal binding (2.5 degrees: 0.879, 5 degrees: 0.86, 7.5 degrees: 0.888, 10 degrees: 0.934). **c** Similarly, there was a subtle increase in the width of the temporal binding window associated with the fusion SIFI with increasing eccentricity (2.5 degrees: 90 ms, 5 degrees: 94 ms, 7.5 degrees: 98 ms, 10 degrees: 99 ms). Curve fits to the individual data indicated that neither of these changes were statistically significant

The same analyses were conducted to assess the impact of visual stimulus eccentricity on the strength of the fusion illusion. Similar to the fission data a two-way repeated-measures ANOVA revealed that while there was a significant main effect of SOA on susceptibility to the fusion illusion, *F*(7, 133) = 66.6, *p* < .001, partial η^2^ = 0.778, BF_10_ = 1.177e+140, there was no significant effect of eccentricity, *F*(3, 57) = 2.58, *p* = .06, partial η^2^ *=* 0.119, BF_10_ = 0.016 (see Fig. 4). The interaction between eccentricity and SOA was not significant, *F*(21, 399) = 1.4, *p* = .112, partial η^2^ = 0.069, BF_10_ = 0.105, suggesting that the impact of SOA on fusion susceptibility does not depend on changes in eccentricity, nor vice versa.

The parameter estimates derived from the Gaussian curve fit to the group-averaged fusion data suggested that the amplitude and width of the temporal binding window associated with the fusion illusion might increase in a systematic fashion with increasing eccentricity of the visual stimulus (see Fig. 4). However, the individual data analysis showed that the effect of visual stimulus eccentricity on the peak amplitude of the temporal binding window failed to reach significance with the fusion illusion, *F*(3, 48) = 1.31, *p* = .282, η^2^ *=* 0.076, showing weak support for the null (BF_10_ = 0.327). Furthermore, eccentricity did not have a statistically significant effect on the standard deviation values derived from the Gaussian curve fits, *F*(3, 48) = 0.49, *p* = .694, η^2^ *=* 0.029, BF_10_ = 0.13.

## Discussion

Contrary to our hypotheses, our results suggest that the strength of the SIFI is not impacted by the retinal eccentricity of the visual flash stimuli. Specifically, manipulating the eccentricity of the visual stimuli had little effect on either the peak amplitude or the width of the temporal binding windows associated with either the fission or fusion variants of the SIFI. This stands in contrast to the idea that the SIFI is the result of direct cortical projections from auditory to visual cortex, which have been shown to predominantly terminate in areas responsible for processing the peripheral visual field (Falchier et al., 2002).

At first glance it might appear that our results are at odds with those from previous studies that showed a difference in the strength of the fission illusion in the fovea and the periphery (Chen et al., 2017; Shams et al., 2001; Tremblay et al., 2007); however, it should be pointed out that we did not include a condition in which the visual stimulus was presented directly to the fovea and therefore did not make a direct comparison between foveal and peripheral responses to the SIFI. Rather, given that there appears to be a consensus that the SIFI is stronger in the peripheral field than the fovea, we sought to test whether the SIFI became progressively larger as the visual stimulus moved further into the periphery, which our results indicated not to be the case. Thus, it appears that while differences exist in susceptibility to the SIFI when comparing foveal and peripheral presentation of the visual stimulus, this effect is similar for several locations in the periphery at least up to 10 degrees from fixation (see Fiebelkorn et al., 2011; Stiles et al., 2018, for other audiovisual phenomena not impacted by visual stimulus eccentricity). Such differences in foveal and peripheral responses might be expected from previous work showing qualitative differences in temporal processing between the fovea and the periphery (Hess & Snowden, 1992; Horiguchi et al., 2009; McKee & Taylor, 1984). It should also be noted that the Gaussian fits to the temporal binding window of the fission SIFI give the impression of a marginal increase in peak amplitude as the visual stimulus is moved further into the periphery (see Fig. 3), a pattern consistent with our *a priori* expectations. However, the Bayes factors associated with the standard and curve fit analyses both provided strong evidence in favour of the null hypothesis (BF_10_ of 0.01 and 0.1, respectively), suggesting that this was not due to study constraints such as those influencing statistical power.

Our finding that the fission SIFI is not modulated by the eccentricity of the visual stimulus speaks against theories suggesting that the SIFI is a result of direct connections from auditory to visual cortices, which have been shown to be more numerous in regions of primary visual cortex and visual area V2 serving peripheral vision (Falchier et al., 2002; Rockland & Ojima, 2003). For example, Falchier et al. (2002) revealed that the number of direct projections from auditory cortex to regions of visual cortex representing central and paracentral regions of the visual field were very low relative to those areas representing the peripheral visual field. In line with this, Chen et al. (2017) explained their finding of a larger fission illusion in the periphery relative to the central field in terms of this enhanced input from auditory cortex in peripheral regions of visual cortex (see Rockland & Ojima, 2003, for a similar argument). However, if these direct corticocortical projections were to give rise to the SIFI, it might be expected that the magnitude of the SIFI would grow monotonically with increasing eccentricity, a hypothesis that our findings do not support. One caveat with this argument is that due to technical reasons imposed by COVID-19 restrictions, the largest eccentricity we could test in the current study was 10 degrees, which is at the lower limit of what Falchier et al. (2002) consider to be the peripheral visual field. However, it should be highlighted that (a) Falchier et al. (2002) reported differences in the number of projections from auditory cortex to area 17 for the eccentricities used in the current study and (b) there was no significant difference between the peak amplitude of the temporal binding windows for the 2.5 and 10-degree eccentricity conditions, *t*(14) = 0.325, *p* = .75, with the associated Bayes factor again providing support for the null hypothesis (BF_10_ = 0.275). Nonetheless, future studies should test further into the peripheral visual field to see if this has any impact on the strength of the SIFI.

Our results also show that the shape of the temporal binding window associated with the fusion SIFI is not impacted by visual stimulus eccentricity. While no previous study to the authors’ knowledge systematically manipulated the eccentricity of the visual stimulus and observed the effect on the SIFI as in the current study, the findings regarding whether the fusion illusion is impacted by position in the visual field have been mixed, with one study showing that the fusion illusion is stronger when the visual stimulus is presented to the fovea (Chen et al., 2017) and the other showing no difference when the stimulus was presented in the central or peripheral visual field (Tremblay et al., 2007). Our results suggest that, if anything, the fusion illusion becomes stronger as it is moved further into the periphery (Fig. 4b); however, we should not read too much into these results given that there was no significant difference in the peak amplitude of the temporal binding window associated with the fusion illusion for the difference eccentricity conditions (albeit the null hypothesis in this case only received weak or “anecdotal” support from the Bayes factor analysis).

Given that no effects of visual stimulus eccentricity were observed on either the fission or fusion illusion, it is reasonable to consider whether our paradigm was sensitive enough to pick up on any differences should they exist. While early studies using the SIFI suggested that it was robust to different contexts and different stimulus parameters (Shams et al., 2000), a number of studies have since shown that both the magnitude and width of the temporal binding associated with the SIFI is subject to change both within (e.g., Andersen et al., 2004; Perez-Bellido et al., 2015; Takeshima & Gyoza, 2013) and between (e.g., Foss-Feig et al., 2010; McGovern et al., 2014; Noel et al., 2016; Setti et al., 2011; Stevenson et al., 2014) groups (as reviewed in Hirst et al., 2020). Furthermore, it has also been demonstrated that the properties of the temporal binding window associated with the SIFI can change in the same individuals following a period of perceptual training (Setti et al., 2014), while other studies show that the magnitude of the SIFI is modulated by expertise (e.g., Bidelman, 2016; Bidelman & Heath, 2018). Together, these findings suggest that the current paradigm should have the requisite sensitivity to pick up on changes in the temporal binding window associated with changes in visual stimulus eccentricity if such effects were present.

Given the public health measures in place due to COVID-19 during the time of data collection, it was not possible to complete the experiment in the laboratory and therefore we could not use an eye-tracker to validate that participants maintained fixation. In lieu of an eye-tracker, several precautions were taken to mitigate the possibility of participants not maintaining a steady fixation. First, a chin-rest was used to help stabilize participants’ heads, and the height of the screen was adjusted for each participant such that fixation cross was presented in their natural eyeline. Second, participants completed practice trials, in which the experimenter assessed each participant’s ability to maintain fixation. Third, the experimenter was present during the experiment and prompted participants to re-fixate on the fixation cross should their gaze shift, although these occurrences were rare. Furthermore, the fully interleaved nature of the trials (across different eccentricities, trial types, and SOAs) ensured it was very difficult for participants to predict the location of the visual stimuli on a trial-to-trial basis. Thus, we are confident that eye movements did not impact on our results. This view is supported by an analysis of the visual-only trials, which shows that participants’ exhibit a reduced sensitivity in discriminating the number of flashes in the absence of auditory stimulation as the stimulus was moved further into the periphery (see Supplementary Fig. 1). These findings are in keeping with previous reports of reduced sensitivity in numerosity judgements when stimuli are presented in the periphery relative to the central visual field (Kumpik et al., 2014; Palomares et al., 2011; Parth & Rentschler, 1984), although it is unclear why this reduced unimodal sensitivity did not produce a larger SIFI, in accordance with reliability-weighting models of multisensory integration (e.g., Ernst & Banks, 2002; see also Kumpik et al., 2014).

In sum, our findings suggest that the eccentricity of the visual stimulus used to elicit the SIFI has little impact on susceptibility to either the fission or fusion variant of this illusion. These results provide important clues as to the neuroanatomical basis of the SIFI, which previous studies had suggested may arise from a predominance of direct connections from the auditory cortex to the parts of the visual cortex responsible for representing the peripheral visual field. While the current results do not support this explanation, future research could examine whether any differences in susceptibility to the SIFI are observed in areas of the periphery beyond 10 degrees from fixation.

## Supplementary Information

ESM 1 (PDF 102 kb)
