# Supplementary material for: The magnitude of the sound-induced flash illusion does not increase monotonically as a function of visual stimulus eccentricity
Source: Atten Percept Psychophys. 2022 May 13;84(5):1689–98. doi: 10.3758/s13414-022-02493-4 (PMC9106326; doi:10.3758/s13414-022-02493-4)
Supplement: Supplementary file 1 — (PDF 102 kb) [file 13414_2022_2493_MOESM1_ESM.pdf]

Supplementary information: The Magnitude of the Sound-Induced Flash Illusion Does Not Increase Monotonically as a Function of Visual Stimulus Eccentricity

Niall Gavin[1], Rebecca J. Hirst[2] & David P. McGovern[1*]

[1] School of Psychology, Dublin City University, Dublin 9, Ireland

[2] School of Psychology and Institute of Neuroscience, Trinity College Dublin, Dublin 2, Ireland

[*]Email: david.p.mcgovern@dcu.ie

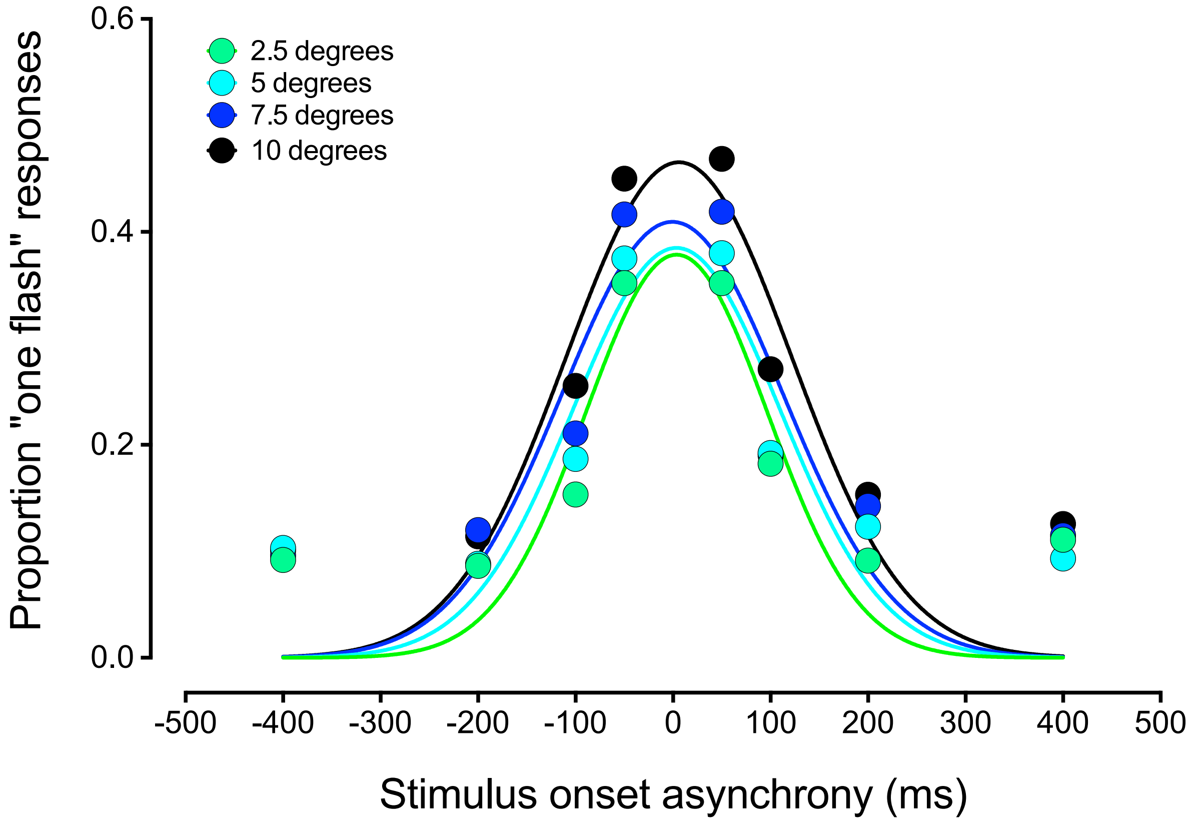

Supplementary Figure 1: Data for two flash, visual-only trials. Participants erroneously reported one flash for a high proportion of trials with short SOAs, with the number of errors increasing as a function of the eccentricity of the visual stimuli.
